# Clinical, Transcriptional and Haplotype Characterization of Recurrent MYBPC3 Splice-Site Variants c.1458-1G>A and c.3331-1G>A Associated with Hypertrophic Cardiomyopathy in Northern Italy

**DOI:** 10.3390/genes17080882

**Published:** 2026-07-28

**Authors:** Carlotta Pia Cristalli, Maria Alessandra Schiavo, Miryam Rosa Stella Foti, Sara Calabrese, Federica Isidori, Alice Margutti, Pierluigi Laricchiuta, Giulia Governatori, Francesco Lai, Vera Uliana, Federico Barocelli, Elia De Maria, Alessandro Fucili, Biagio Sassone, Giulia Parmeggiani, Enrica Perugini, Camilla Lucca, Francesca Cappuccini, Laura Pezzoli, Maria Iascone, Maria Piane, Giovanni Vitale, Claudio Graziano, Rita Selvatici, Alessandra Ferlini, Maddalena Graziosi, Elena Biagini, Daniela Turchetti, Francesca Gualandi, Cesare Rossi

**Affiliations:** 1 SSD Biologia e Medicina Molecolare, IRCCS Azienda Ospedaliero-Universitaria di Bologna, 40138 Bologna, Italycesare.rossi@aosp.bo.it (C.R.); 2 Cardiology Unit, Cardiac Thoracic and Vascular Department, IRCCS Azienda Ospedaliero-Universitaria di Bologna, 40138 Bologna, Italy; 3 European Reference Network for Rare, Low Prevalence and Complex Diseases of the Heart (ERN GUARD-Heart), 1105 Amsterdam, The Netherlands; 4 Unit of Medical Genetics, Department of Medical Sciences and Department of Mother and Child, University Hospital S. Anna Ferrara, 44121 Ferrara, Italy; 5 Medical Genetics Unit, IRCCS Azienda Ospedaliero-Universitaria di Bologna, 40138 Bologna, Italygiulia.governatori6@unibo.it (G.G.);; 6 Unit of Cardiology, Bentivoglio Hospital, 40010 Bentivoglio, Italy; 7 Medical Genetics, University Hospital of Parma, 43126 Parma, Italy; 8 Unit of Cardiology, University Hospital of Parma, 43126 Parma, Italy; 9 Cardiology Unit, Ramazzini Hospital, Carpi, 41012 Modena, Italy; 10 Cardiology Department, University Hospital S.Anna Ferrara, 44121 Ferrara, Italy; 11 Cardiology Unit, SS.ma Annunziata Hospital, Department of Translational Medicine, University of Ferrara, 44121 Ferrara, Italy; 12 Medical Genetics Unit, Department of Clinical Pathology, AUSL Romagna, 48121 Cesena, Italy; 13 Cardiology Unit, Maggiore Hospital, 40138 Bologna, Italy; 14 Laboratory of Medical Genetics, ASST Papa Giovanni XXIII, 24127 Bergamo, Italy; 15 Sant’Andrea University Hospital, 00189 Rome, Italy; 16 Department of Clinical and Molecular Medicine, Sapienza University of Rome, 00185 Rome, Italy; 17 Cardiology Unit, Ospedale Santa Maria Della Scaletta, 40026 Imola, Italy

**Keywords:** *MYBPC3*, founder mutation, hypertrophic cardiomyopathy

## Abstract

**Background:** Founder mutations in *MYBPC3* may contribute substantially to the genetic burden of hypertrophic cardiomyopathy (HCM) and provide important insights into genotype–phenotype correlations and population-specific disease mechanisms. In this study, we investigated two recurrent canonical splice-site variants, *MYBPC3* c.1458-1G>A and c.3331-1G>A, identified in patients with HCM from the Emilia-Romagna region of Northern Italy. **Methods:** Ninety-one unrelated patients with HCM carrying either *MYBPC3*c.1458-1G>A or c.3331-1G>A were analyzed. Haplotype reconstruction was performed to assess a possible founder effect and estimate the approximate age of the shared ancestral allele. Functional characterization was carried out by RNA sequencing of myocardial tissue to evaluate the impact of the variants on splicing. Clinical and phenotypic data were compared with those of carriers of other truncating *MYBPC3* variants or pathogenic variants in other sarcomeric genes. **Results:** Both variants, classified as pathogenic according to ACMG criteria, shared a conserved variant-specific core haplotype. Founder age was estimated at approximately 10.5 generations (~262 years), consistent with a regional founder effect. RNA sequencing demonstrated aberrant splicing for both variants, resulting in premature termination codons and presumably subsequent nonsense-mediated mRNA decay. Clinically, carriers showed delayed disease onset, with a mean onset in the fifth decade of life, and a comparatively milder phenotype, including a lower incidence of sudden cardiac death, than patients carrying other truncating *MYBPC3* variants or pathogenic variants in other sarcomeric genes. Both variants exhibited partial penetrance (approximately 63–67%) and age-dependent variable expressivity. **Conclusions:** *MYBPC3* c.1458-1G>A and c.3331-1G>A represent novel founder alleles associated with HCM in Northern Italy. Identification of these locally prevalent variants improves molecular diagnosis, family screening, and supports the development of variant-targeted therapeutic approaches.

## 1. Introduction

Hypertrophic cardiomyopathy (HCM) is a cardiac disorder characterized by increased left ventricular (LV) wall thickness, with or without concomitant right ventricularhypertrophy (RVH), that cannot be explained solely by abnormal loading conditions [1]. HCM is the most common inherited cardiomyopathy, with an estimated prevalence of 0.2–0.5% worldwide [2]. Approximately half of cases are monogenic disorders with autosomal dominant inheritance, incomplete penetrance, and variable expressivity. The condition is genetically heterogeneous: variants in at least 29 genes with definitive, strong, or moderate evidence of causation for HCM or isolated left ventricular hypertrophy (LVH), including sarcomere and sarcomere-associated genes, or ion channel genes, have been implicated [3]. The clinical manifestations and prognosis of HCM are highly variable. Some carriers remain asymptomatic, while others develop advanced disease with severe heart failure (HF) or sudden cardiac death (SCD) [4]. Pharmacological and surgical interventions are primarily aimed at symptom relief and slowing disease progression. Genetic testing plays an important role in the management of patients and their relatives, but the diagnostic yield (detection of pathogenic/likely pathogenic variants) ranges from 28 to 40% [5]. Although high-throughput sequencing technologies have expanded the number of genes interrogated, this has not translated into substantially higher yields. Many variants are private to individual families, precluding robust genotype–phenotype correlations [6].

The two most frequently mutated genes in HCM are *MYBPC3* and *MYH7*, which together account for 70–80% of genetically positive diagnoses [7]. *MYBPC3* (OMIM #600958), located on chromosome 11p11.2 (GRCh38/hg38: chr11:47,331,406–47,352,702; ENST00000545968, NM_000256.3), encodes cardiac myosin-binding protein C (cMyBP-C). The gene spans over 21 kb and contains 35 exons. cMyBP-C, a multi-modular sarcomeric protein essential for contractile regulation, localizes to the C-zone of the A-band and forms transverse doublets in the cross-bridge region [8]. These structural features facilitate binding to myosin and actin filaments, thereby contributing to sarcomeric stability, force generation, and calcium sensitivity. *MYBPC3* pathogenic variants drive cardiomyocyte hypertrophy, sarcomeric disarray, and interstitial fibrosis [4,7].

As of May 2026, 1042 pathogenic or likely pathogenic *MYBPC3* variants have been reported in ClinVar. Most pathogenic variants cause premature translation termination, and likely trigger nonsense-mediated mRNA decay (NMD); the resulting haploinsufficiency reduces functional protein levels to approximately 50–70% of normal [8]. Additionally, missense variants and small in-frame indels accounting for roughly 15% of *MYBPC3*-related cases produce full-length but dysfunctional proteins that destabilize sarcomeric function.

Founder *MYBPC3* variants have been identified in several populations (Iceland, the Netherlands, Finland, Japan, South Asia, France), where they represent a substantial fraction of HCM cases. All these founder alleles are truncating variants that result in shortened cMyBP-C lacking critical phosphorylation motifs and/or major sarcomeric binding domains [8]. In Italy, founder variants have been described in the Veneto and Tuscany regions. The c.912_913del (p.Phe305ProfsTer27) variant in exon 11 was found in 19.5% of HCM probands in Veneto [9], whereas the c.772G>A (p.Glu258Lys) variant in exon 6 was identified in 14% of probands in Tuscany [10]. These founder variants frequently show incomplete penetrance and variable expressivity, with phenotypes ranging from isolated LVH to arrhythmic events and increased risk of SCD. More recently, the c.2309-2A>G variant has been reported as part of a common haplotype in Central Italy, with penetrance influenced by age and sex. Male carriers show earlier onset, higher penetrance, and more severe disease [11]. The aim of this study is to characterize in detail the clinical phenotype, longitudinal outcomes, and management of individuals carrying two specific *MYBPC3* splice-altering variants (c.1458-1G>A and c.3331-1G>A) in a cohort of Italian HCM patients. In addition, we aimed to confirm, at the molecular level, that the two variants have a disruptive effect on protein synthesis, as opposed to possibly milder outcomes such as altered splicing with preservation of the reading frame.

## 2. Materials and Methods

### 2.1. Study Populations

The protocol conforms to the ethical norms and standards in accordance with the Declaration of Helsinki. All subjects, or their legal representatives, were provided with written informed consent for genetic analysis and publication of results. Patients were recruited through Cardiology and Medical Genetics Units from eight hospitals in the Emilia-Romagna region (Italy) between 2018 and 2024. Each participating centre completed a standardized electronic patient’s record with personal and family history, clinical features, and instrumental cardiovascular findings. The study group included 99 subjects carrying either of two recurrent splice-site variants in the *MYBPC3* gene (c.1458-1G>A and c.3331-1G>A). The study cohort used for clinical comparisons consisted of 91 out of 99 patients (8 were excluded because still asymptomatic due to their young age). A total of 26 probands and 11 relatives carried the c.3331-1G>A variant, whereas 42 probands and 20 relatives carried the c.1458-1G>A variant. Three control populations (A, B, and C) were included, comprising a total of 129 patients with both a clinical diagnosis of HCM and a positive genetic test. The control populations were collected in the same time frame as our study cohort and underwent a similar follow-up. Control population A (n = 38) included individuals carrying a missense *MYBPC3* variant classified as likely pathogenic (class 4) or pathogenic (class 5). Control population B (n = 46) included individuals carrying truncating *MYBPC3* class 4 or 5 variants, whereas control population C (n = 45) comprised individuals with pathogenic variants in other sarcomeric genes (*TNNI3*, *TNNT2*, *MYH7*, *MYL3*), according to ACMG guidelines.

### 2.2. Cardiological Assessment

HCM was diagnosed by the presence of a hypertrophied left ventricle (maximum left ventricular wall thickness [MLVWT] ≥15 mm in adult index patients or ≥13 mm in adult relatives) in the absence of other cardiac or systemic conditions capable of causing LVH. The evaluation of probands and relatives carrying the MYBPC3 variants included medical history, physical examination, 12-lead ECG, and M-mode, two-dimensional, and Doppler echocardiography. Cardiac magnetic resonance (CMR) was not performed in all patients; therefore, these data were excluded from further evaluation. Regarding the control populations (A, B, and C), the same clinical and instrumental data were collected.

### 2.3. Genetic Analysis

#### 2.3.1. Next-Generation Sequencing (NGS)

Genetic testing was performed using targeted NGS panels. All participants underwent analysis of at least the eight canonical HCM genes (*MYBPC3*, *MYH7*, *TNNT2*, *TNNI3*, *TPM1*, *MYL2*, *MYL3*, and *ACTC1*). Variants identified by NGS were filtered based on sequencing quality, read depth, and population allele frequency, applying a minor allele frequency (MAF) threshold of ≤0.01. Variant classification followed ACMG guidelines using the five-tier framework (pathogenic, likely pathogenic, variant of uncertain significance, likely benign, benign) [12] and, when available, gene-specific criteria according to ClinGen (https://www.clinicalgenome.org/, accessed on 27 May 2026). In carriers of the *MYBPC3* c.1458-1G>A or c.3331-1G>A variants, no additional pathogenic or likely pathogenic variants were detected. The presence of these variants and their segregation within affected families were confirmed by Sanger sequencing using specifically designed primers.

#### 2.3.2. RNA Sequencing (RNA-Seq)

Freshly frozen myocardial tissues obtained from myectomy of 3 unrelated HCM patients were used for RNA-based analyses. The patients consented to the use of the collected tissue for further analysis and to the scientific publication of the results in aggregated and anonymized form. Total RNA was extracted using the QIAsymphony RNA Kit and the RNeasy Mini Kit (QIAGEN, Hilden, Germany) according to the manufacturers’ protocols. Libraries were prepared using the Illumina Stranded mRNA Prep kit (Illumina, San Diego, CA, USA), and massively parallel sequencing was performed on a NovaSeq 6000 system (SP flow cell) with 100 bp paired-end reads. Reads were aligned to the human reference genome GRCh38/hg38, and variant calling was performed using DRAGEN RNA (v.4.2.7). Aberrant splicing events were identified using FRASER (v.2.0) [13], while haploinsufficiency was evaluated by comparing the ratio of *MYBPC3* to *MYH7* transcripts per million (TPM) in patients carrying the c.1458-1G>A or c.3331-1G>A variants versus controls (HCM patients with variants in sarcomeric genes other than *MYBPC3*) [14].

### 2.4. Haplotype Analysis

Haplotype analyses were conducted using five sequence-tagged site (STR) markers: D11S1993 (Chr11:43,588,370–43,588,779), D11S986 (Chr11:44,700,564–44,700,838), D11S4174 (Chr11:45,236,466–45,236,824), D11S1344 (Chr11:46,145,309–46,145,675), and D11S1395 (Chr11:54,736,244–54,736,497) in 42 index cases and 6 family members. These markers, selected from the UCSC Genome Browser (GRCh38/hg38), span approximately 11.15 Mb of chromosome 11. Forward primers were labelled with 6-fluorescein amidite (FAM), and PCR products were analyzed with an internal sizing standard (GeneScan™ 500 LIZ) using an ABI 3730xl DNA Analyzer (Applied Biosystems, Foster City, CA, USA). Amplicon sizes were determined using Peak Scanner Software v1.0. Haplotype reconstruction was performed manually from the observed STR genotypes. Whenever family members were available, segregation was used to determine the phase and to confirm the association between each *MYBPC3* variant and its corresponding haplotype. The minimal conserved haplotypic segment shared among apparently unrelated carriers of each variant was subsequently identified. The time to the most recent common ancestor (TMRCA) was estimated using a linkage disequilibrium (LD) decay approach based on the extent of the shared ancestral haplotype. Physical distances (GRCh38/hg38) were converted into genetic map distances assuming an average recombination rate of 1.0 cM/Mb and a generation interval of 25 years. The resulting estimate should be interpreted as an order-of-magnitude approximation of the timing of the founder events rather than as a formal demographic inference.

### 2.5. Statistical Analysis

Continuous variables are presented as median (interquartile range), and categorical variables are expressed as percentages. Statistical analyses were performed using SPSS (SPSS Statistics 25) for Windows. Categorical variables were compared using Fisher’s exact test due to small sample sizes in some cohorts. Continuous variables were compared using the Mann–Whitney U test, given the non-normal distribution and small sample sizes. A *p*-value <0.05 was considered statistically significant.

## 3. Results

### 3.1. Frequency of the MYBPC3 c.1458-1G>A and c.3331-1G>A Splicing Variants

Between January 2018 and December 2024, we identified 62 carriers of the *MYBPC3* c.1458-1G>A variant, of whom 57 were diagnosed with HCM and 5 were phenotype-negative. Additionally, 37 patients carried the c.3331-1G>A variant, with 34 affected by HCM. Together, these two *MYBPC3* variants account for more than 50% of genetically diagnosed HCM cases with a pathogenic or likely pathogenic variant in a relevant gene in ourregional cohort. Carriers of either c.1458-1G>A or c.3331-1G>A were predominantly probands presenting with HCM, with several additional cases identified through family screening. Detailed family history spanning at least three generations was available for 35 out of 42 c.1458-1G>A cases and 18 out of 26 c.3331-1G>A cases. To estimate the frequency of the two variants in the general local population, we queried an in-house Whole Exome Sequencing (WES) database comprising 2523 individuals referred to our centre for indications other than cardiomyopathy. The c.1458-1G>A variant was present in two individuals, both of whom were subsequently found to have HCM, whereas the c.3331-1G>A was absent from the cohort.

### 3.2. Pedigree Analysis of MYBPC3 c.1458-1G>A Carriers

**Figure 1:**
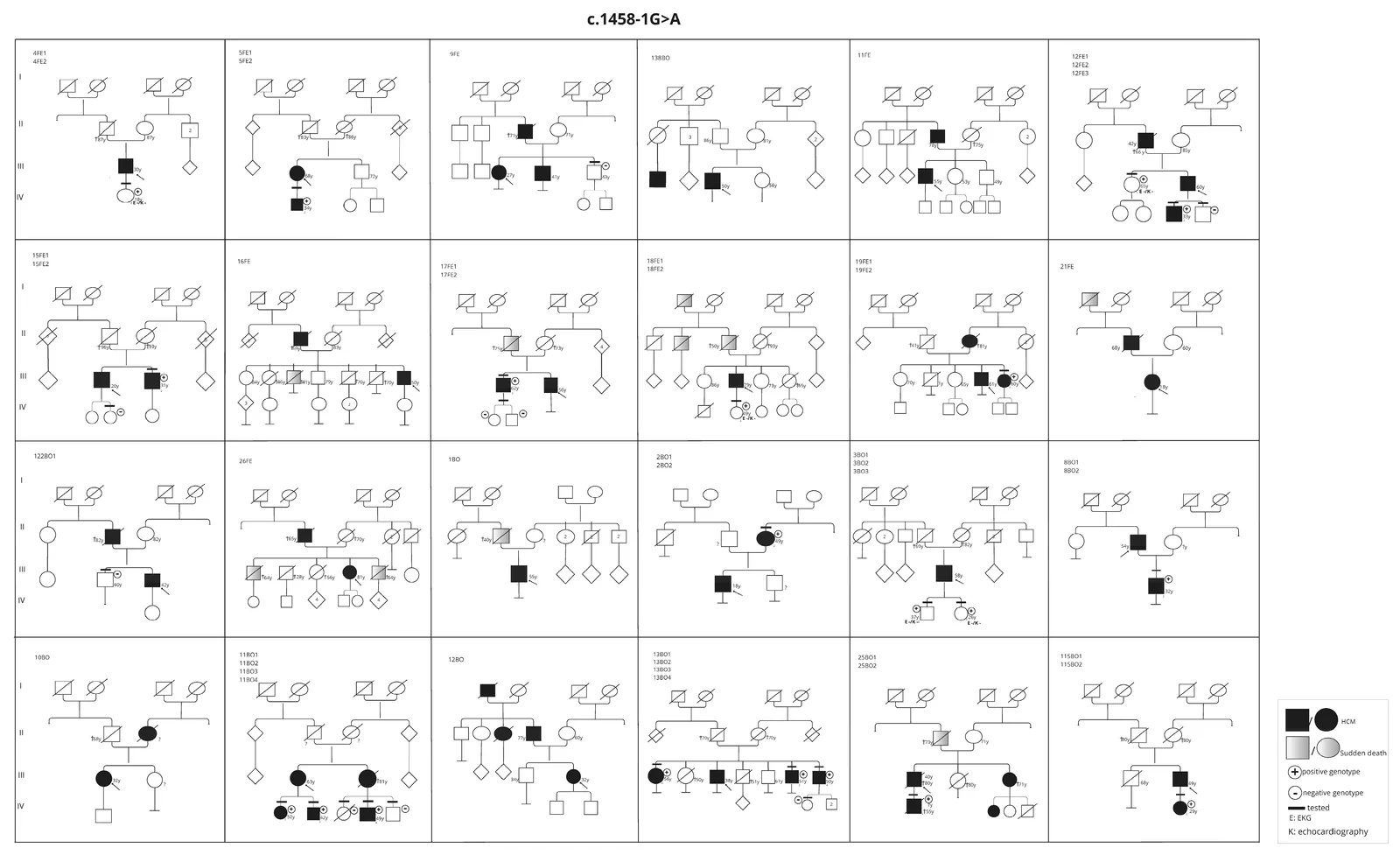
c.1458-1G>A pedigrees.

Recurrence of HCM was observed in 20 of 35 families harbouring the c.1458-1G>A variant. Sudden cardiac death was documented in 7 of these families. Among 20 genotype-positive relatives, 15 manifested an HCM phenotype, yielding an estimated penetrance of 66.7%. Of the 5 unaffected carriers, 3 were last evaluated at an age younger than the median age at diagnosis within the cohort (Figure 1).

### 3.3. Pedigree Analysis of MYBPC3 c.3331-1G>A Carriers

**Figure 2:**
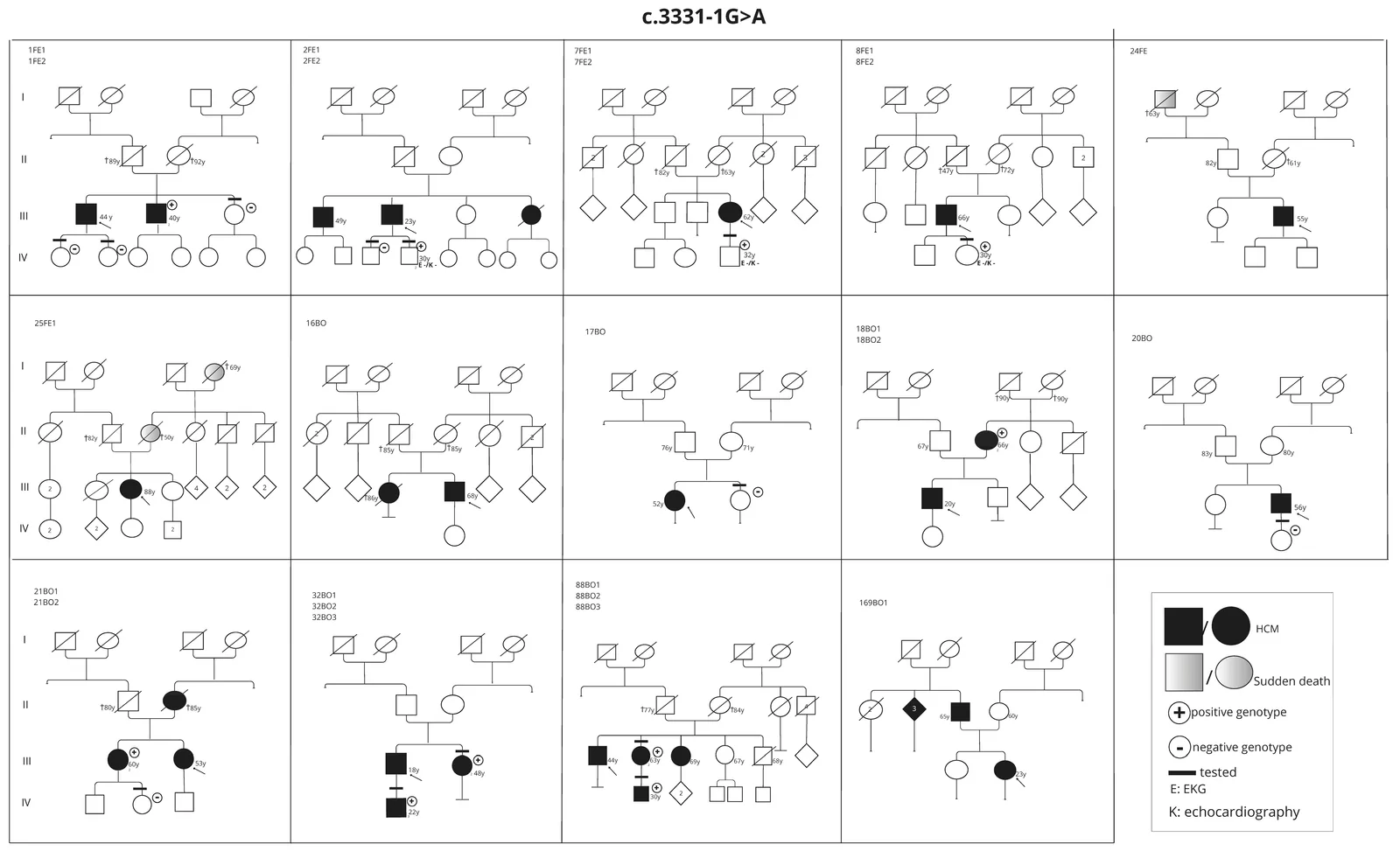
c.3331-1G>A pedigrees.

Recurrence of the HCM phenotype was observed in 7 of 18 families carrying the c.3331-1G>A variant. Familial SCD was documented in 2 families. Among 11 genotype-positive relatives, 8 manifested an HCM phenotype, corresponding to an estimated penetrance of 62.5%. The 3 unaffected carriers had last been evaluated at age 30 years, younger than the median age at diagnosis in the cohort (Figure 2).

### 3.4. Impact of the MYBPC3 c.1458-1G>A and c.3331-1G>A Variants on Splicing

**Figure 3:**
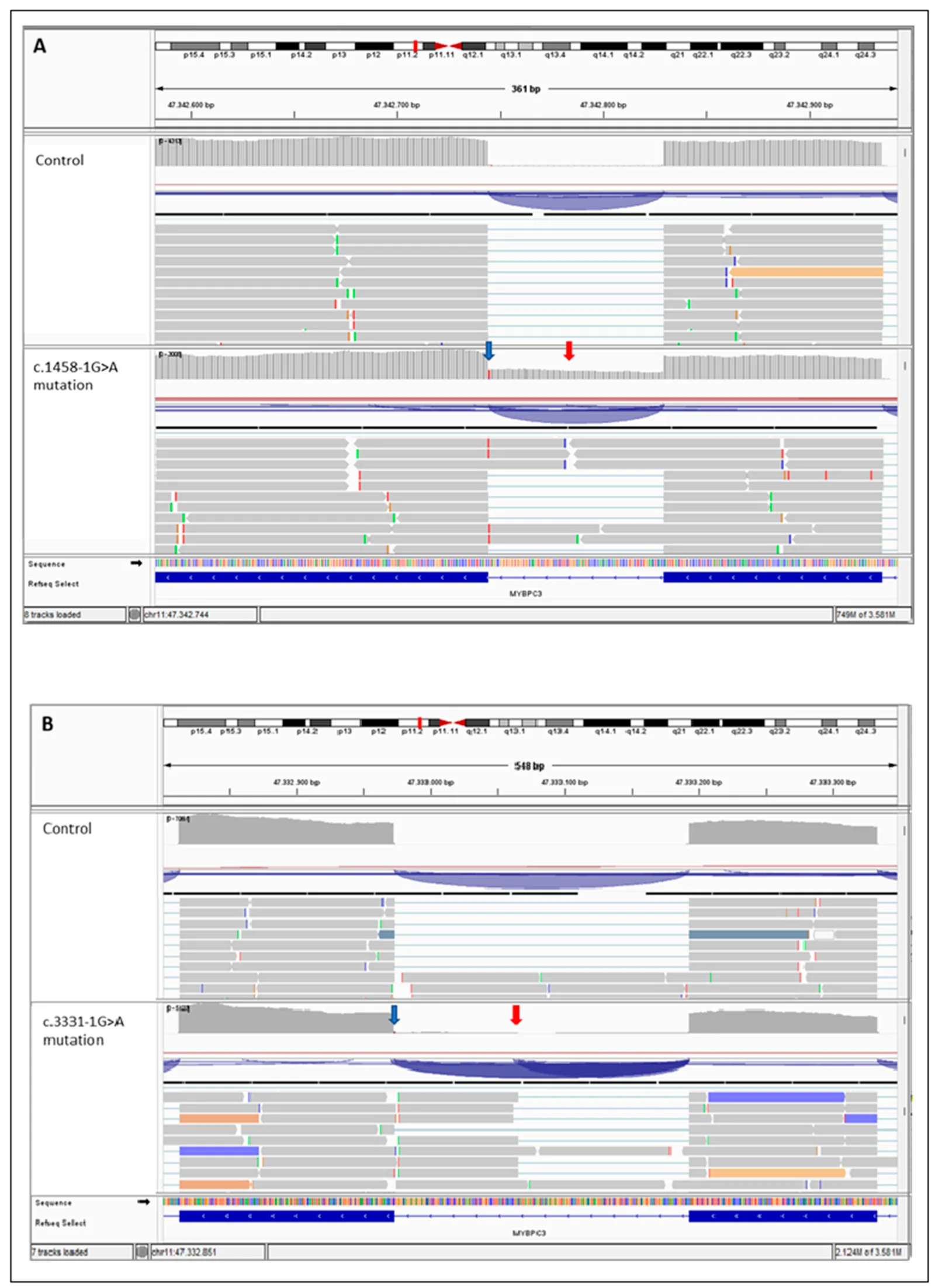
RNA-seq on myocardial tissue of c.1458-1G>A (panel (**A**)) and c.3331-1G>A (panel (**B**)) carriers. Both panels show the IGV visualization of RNA sequencing for a wild-type control and a mutated case; The blue arrows indicate the position of the two variants, while the red arrows show the coverage (panel (**A**,**B**)) and reads resulting from aberrant splicing (panel (**B**)). For both mutations, the coverage of the putative intronic region is lower than it would be if wild-type and mutant transcripts were present in equal amounts. The same applies to the number of aberrant splicing events, suggesting negative selection of the mutant transcript.

RNA-seq analysis of myocardial tissue from one carrier of the c.3331-1G>A variant revealed aberrant splicing, including retention of 89 or 92 base pairs from intron 30 presumably through activation of cryptic splice donor sites. In both instances, inclusion of partial intronic sequence introduces a frameshift and generates a premature stop codon at the protein level. In contrast, analysis of myocardial tissue from two carriers of the c.1458-1G>A variant demonstrated retention of the entire intron 16, which likewise results in frameshift and introduction of a premature stop codon. RNA-seq data enabled detailed coverage analysis of the retained intronic regions relative to the transcript from the normal *MYBPC3* allele (Figure 3A,B). The analysis of the relative expression of the *MYBPC3* transcript—performed by comparing the ratio of *MYBPC3* to *MYH7* TPMs in mutated cases versus controls—showed reduced *MYBPC3* expression in cases with the two mutations, consistent with negative selection of aberrant transcripts. This finding is supported both by the coverage of the retained intronic region and the number of aberrant splicing events, and by the allelic imbalance of other SNPs present in the transcript. The lower relative abundance of aberrant transcripts suggests they undergo degradation, presumably through NMD.

### 3.5. The c.1458-1G>A and c.3331-1G>A Variants Reside on Shared Haplotypes

**Figure 4:**
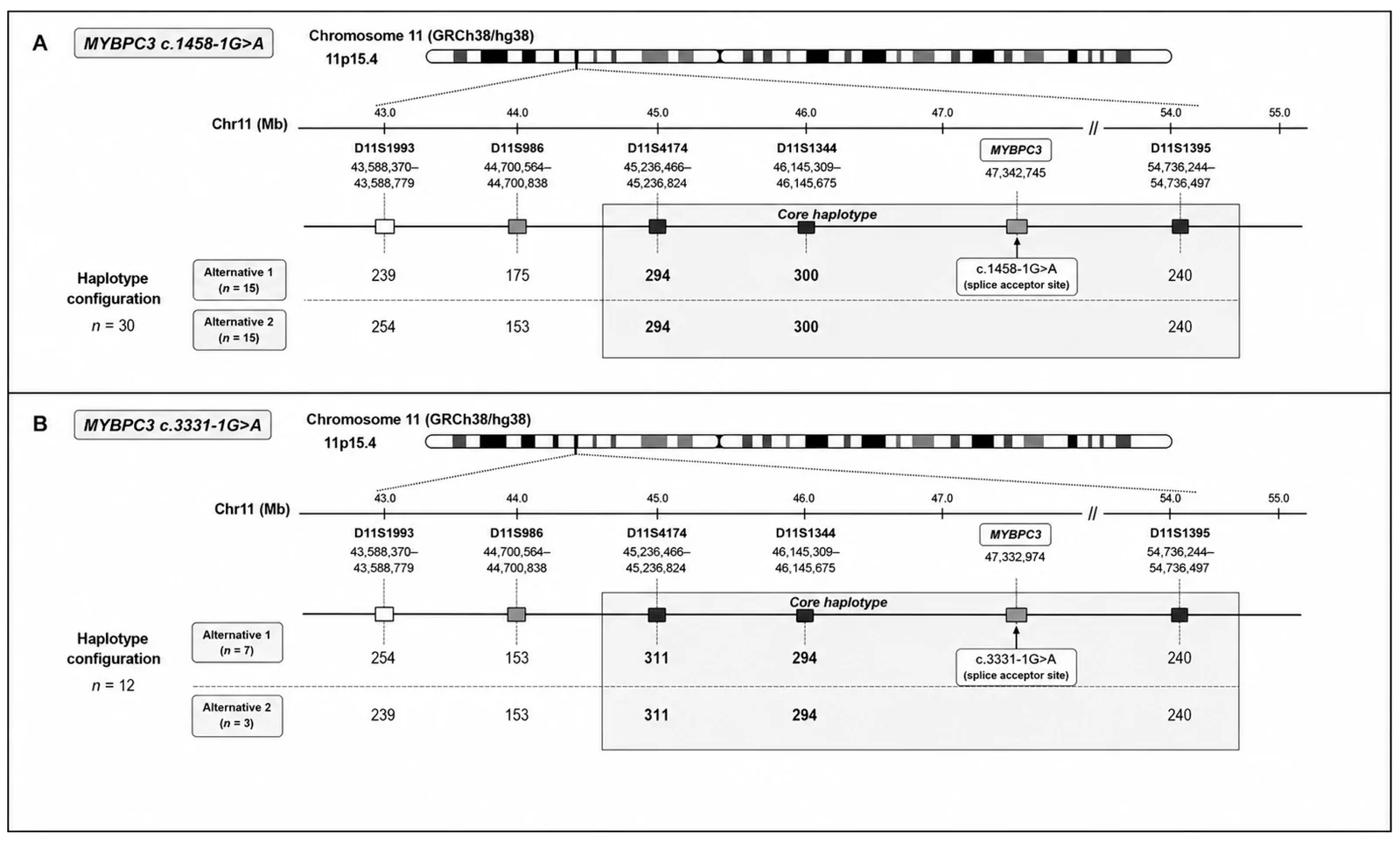
Haplotype configurations associated with *MYBPC3* splice-site variants. (**A**) Schematic representation of the *MYBPC3* genomic region on chromosome 11p15.4 (GRCh38/hg38) associated with the c.1458-1G>A splice-site variant. The core haplotype, defined by the shared alleles at D11S4174, D11S1344, and D11S1395, is highlighted in grey. Two alternative haplotype configurations were identified among carriers of the variant. (**B**) Schematic representation of the *MYBPC3* genomic region associated with the c.3331-1G>A splice-site variant. The same set of informative microsatellite markers and genomic coordinates is displayed. The shared core haplotype (D11S4174, D11S1344, and D11S1395) is highlighted in grey. Two alternative haplotype configurations were observed among carriers.

Haplotype analysis revealed a shared genomic segment among all index cases (n = 42). The investigation included five STR markers (D11S1993, D11S986, D11S4174, D11S1344, D11S1395). Genotyping of all five STR markers demonstrated that each splice-site variant resides on a distinct conserved haplotypic background shared among all of its carriers (Figure 4). Specifically, all carriers of c.1458-1G>A shared an identical core haplotype encompassing the markers D11S4174, D11S1344, and D11S1395, while all carriers of c.3331-1G>A shared a different, but likewise conserved, core haplotype across the same markers, spanning approximately 9.5 Mb around the *MYBPC3* locus. In contrast, the more centromeric markers D11S986 and D11S1993 segregated into two recurrent haplotypic configurations with comparable frequencies among carriers of each variant (Supplementary Tables S1 and S2). These findings suggest that an early ancestral recombination event upstream of each conserved core haplotype generated two related sub-haplotypes that subsequently spread within the population (Figure 4).

All probands were born and/or currently reside in two neighbouring provinces of the Emilia-Romagna region in Italy, with a combined population of approximately 1.3 million inhabitants, further supporting a local founder effect for both variants. Based on the extent of the conserved ancestral haplotype and assuming an average recombination rate of 1.0 cM/Mb, the time to the most recent common ancestor (TMRCA) was estimated at approximately 10.5 generations, corresponding to about 262 years assuming a generation interval of 25 years. This estimate should be interpreted as an approximate indication of the timing of the founder events rather than as a formal demographic inference. The conservation of the extended haplotypic background among apparently unrelated carriers supports a shared ancestral origin for each variant, consistent with founder mutations rather than independent recurrent mutational events.

### 3.6. Cardiac Phenotype and Clinical Outcomes of Patients Carrying the c.1458-1G>A or c.3331-1G>A Variants

When comparing the two study cohorts, no significant differences were observed in baseline clinical or echocardiographic characteristics, nor in the overall burden of cardiovascular events during follow-up (Table 1). The cohort of 57 patients carrying the c.1458-1G>A variant had a median age at diagnosis of 49 [34–56] years; 77% were male. With regard to HCM phenotype, 19% had an obstructive form (14% LVOT obstruction, 5% midventricular obstruction), and 4% had an apical HCM phenotype. At first evaluation at the referral centre, median maximum wall thickness was 19 [16–22] mm and left atrial diameter 42 [36–48] mm. Only 1 patient (2%) presented with an end-stage phenotype (LVEF <50%), 3 (5%) had moderate or severe mitral regurgitation, and 3 (5%) had a restrictive filling pattern. Over a median follow-up of 9.4 [2.8–14] years, atrial fibrillation occurred in 14 patients (25%), 18 (32%) underwent ICD implantation, and 2 (4%) required septal myectomy. Major ventricular arrhythmias (sustained ventricular tachycardia, ventricular fibrillation, or appropriate ICD therapy) were recorded in 3 patients (5%), while end-stage evolution (LVEF <50%) was documented in 6 (11%). Three patients (5%) died of cardiovascular causes: 2 due to advanced heart failure and 1 from sudden cardiac death. The cohort of 34 patients carrying the c.3331-1G>A variant had a median age at diagnosis of 48 [23–61] years; 63% were male. An obstructive phenotype was observed in 26% (20% LVOT obstruction, 6% midventricular obstruction), and 9% had an apical variant. At baseline evaluation, median maximum wall thickness was 19 [16–23] mm and left atrial diameter 42 [40–48] mm. Three patients (9%) had an end-stage phenotype (LVEF <50%), 3 (9%) had moderate or severe mitral regurgitation, and 1 (3%) had a restrictive filling pattern. Over a median follow-up of 8.5 [2.7–15] years, atrial fibrillation was documented in 9 patients (26%), 11 (34%) received ICD implantation, and 1 (4%) underwent septal myectomy. Major ventricular arrhythmias occurred in 4 patients (11%), end-stage evolution (LVEF <50%) was observed in 14%, and 2 patients (6%) underwent heart transplantation. Three patients (9%) died of cardiovascular causes: 1 due to advanced heart failure, 1 from stroke, and 1 from SCD.

### 3.7. Comparisons of HCM c.1458-1G>A and c.3331-1G>A Patients with Control Cohorts A and B: Other Truncating and Missense MYBPC3Pathogenetic Variants

No significant differences were observed when comparing patients with the c.1458-1G>A or c.3331-1G>A variants to those carrying non-truncating *MYBPC3* pathogenetic variants (control group A). Carriers of the c.1458-1G>A or c.3331-1G>A variants were significantly older at the time of diagnosis or first clinical evaluation and had a family history of sudden cardiac death compared with patients with other truncating *MYBPC3* variants (control group B) (Table 2 and Table 3).

### 3.8. Comparisons of HCM c.1458-1G>A and c.3331-1G>A Patients with Control Cohorts C: Other Sarcomeric Pathogenetic Variants

HCM patients carrying either the c.1458-1G>A or c.3331-1G>A variant were older at diagnosis and had a family history of sudden cardiac death compared with patients harbouring mutations in other sarcomeric genes. Myectomy was also less frequently required in c.1458-1G>A or c.3331-1G>A patients. No significant differences were observed regarding other clinical and echocardiographic features or cardiovascular outcomes, in either comparison (Table 2 and Table 3).

## 4. Discussion

Founder mutations in *MYBPC3* have been previously described across different populations, supporting the notion that recurrent ancestral events contribute substantially to the genetic burden of HCM [11].

In the present study, we identified two canonical splice-site variants in the *MYBPC3* gene, c.1458-1G>A and c.3331-1G>A, as founder mutations in the Emilia-Romagna region of Italy. The interpretation of these variants as founder mutations is supported by the convergence of several observations, including their recurrence in apparently unrelated families from a geographically restricted area, the presence of variant-specific conserved core haplotypes, and the limited haplotypic diversity observed outside the shared ancestral region, consistent with historical recombination events. Both variants, classified as pathogenic (class 5) according to ACMG criteria, were consistently associated with two shared core haplotypes with an estimated age of approximately 10.5 generations (~262 years), suggesting a relatively recent origin from a common ancestor. The geographic clustering of all probands within two neighbouring provinces further supports a strong founder effect. Notably, both variants were previously reported as private pathogenic *MYBPC3* alleles in large patient cohorts, without evidence of recurrence or correlation with clinical outcomes [15,16,17,18,19,20,21].

From a clinical standpoint, patients exhibited a less aggressive phenotype, similar between the two variants and milder than that observed in individuals carrying truncating mutations in the same gene or in other sarcomeric genes. Based on quite comprehensive clinical data, statistically significant parameters included a later age at onset (shifting toward the fifth decade of life), consistent with the age at first evaluation in the reference centre, and a lower incidence of sudden cardiac death (SCD) compared with control populations. Moreover, during follow-up, the occurrence of adverse cardiac events (SCD or fatal arrhythmic events) did not differ significantly from either control cohort analyzed in this study.

The canonical splice-site location of both variants supports a deleterious effect on *MYBPC3* protein expression; in principle, though, splice-site variants could have a lesser impact if they lead to the activation of cryptic splice sites that maintain the transcript reading frame and result in limited insertions or deletions of the protein sequence, generating a (partially) functional allele. Given the very high frequency of the two variants in our HCM patient cohort, we were concerned that this might be the case and wanted to assess the actual functional consequences of each variant at the molecular level. RNA sequencing on myocardial tissue from HCM patients revealed that c.1458-1G>A causes complete retention of intron 16 and generates a frameshift and a truncated protein due to a premature termination codon within the retained intron. The c.3331-1G>A was characterized by a more complex pattern, including retention of 89 or 92 bp from intron 30 and ensuing frameshift and premature termination. These findings provide direct mechanistic evidence supporting the pathogenic role of both variants. The altered transcripts showed low coverage of the retained regions and a lower relative expression, suggesting degradation via nonsense-mediated mRNA decay (NMD). This mechanism is consistent with the established pathogenic model for most truncating and splicing *MYBPC3* variants, in which aberrant transcripts are eliminated, resulting in haploinsufficiency rather than dominant-negative protein production. Furthermore, Glazier et al. described an allelic imbalance resulting in preferential expression of the wild-type allele, which could partially compensate for the loss-of-function effect and contribute to a milder phenotype, as observed in our cases [22]. In our cohort, both the c.1458-1G>A and c.3331-1G>A splice variants demonstrated clear familial recurrence of HCM and segregation with disease, sometimes accompanied by cases of SCD, strongly supporting their pathogenic role. To furthersupport a causative role of these variants in HCM, we screened a regional cohort of 2523whole-exome sequencing (WES) cases referred to our centre for various indications. No individuals carried the c.3331-1G>A variant, whereas two cases were found to harbour the c.1458-1G>A variant, both in a syndromic context that included HCM among other features. The estimated penetrance was similar between the two variants (66.7% for c.1458-1G>A and 62.5% for c.3331-1G>A), consistent with previous reports for other *MYBPC3* splice-site variants [23]. Some of the genotype-positive relatives remained asymptomatic; however, most had not yet reached the median age at diagnosis observed in our cohort, suggesting that penetrance is likely underestimated and may increase with age. This observation reflects the well-known age-dependent expression of *MYBPC3* variants, in which phenotypic manifestations typically emerge during adulthood and progress over time [24]. The familial recurrence of HCM, together with the observed age-dependent penetrance and variable phenotypic expression, mirrors the clinical behaviour described for other pathogenic *MYBPC3* variants. These results highlight the importance of systematic family evaluation and longitudinal follow-up of carriers, as disease expression may manifest later in life. The relevance of our findings is further underscored by comparison with other *MYBPC3* founder mutations. Most notably, the splice-site variant c.2309-2A>G has recently been established as a founder allele in Central Italy. In a multicentre cohort of over 5000 HCM patients, approximately 12% of probands carrying pathogenic *MYBPC3* variants harboured c.2309-2A>G, which segregated with a unique 5.2 Mb haplotype of common ancestral origin. The identification of c.1458-1G>A and c.3331-1G>A as novel Italian founder variants extends the spectrum of *MYBPC3* founder effects beyond the well-documented c.2309-2A>G, as well as c.25_27delGAG in South Asia and c.772G>A in Northern Europe. Compared with these older and geographically widespread alleles, the variants described here are of more recent origin and currently confined to a smaller population cluster in Northern Italy. This observation highlights the contribution of region-specific founder events to the genetic burden of HCM and underscores the importance of considering local population genetics in both diagnostic and research contexts. The recognition of founder variants carries important implications for molecular diagnosis, cascade screening, and clinical management, particularly in regions where such alleles account for a substantial proportion of genetic diagnoses. Furthermore, in the context of emerging gene therapy approaches for HCM, ancestrally inherited and regionally enriched variants may represent attractive targets for variant-specific therapeutic interventions, with the potential to benefit larger patient groups compared with private mutations. Although our haplotype and functional analyses strongly support a founder effect and a causative role in HCM, the cohort’s geographic restriction may limit the generalizability of our findings. Moreover, the clinical characteristics, penetrance estimates, and disease severity observed in our cohort may partly reflect the specific genetic background of the studied population, including the influence of local genetic modifiers. Larger multicentre studies will be necessary to confirm penetrance estimates and clinical outcomes associated with these variants across broader populations.

## 5. Conclusions

This study identifies c.1458-1G>A and c.3331-1G>A as novel *MYBPC3* founder variants in Northern Italy, characterized by a shared haplotype of recent ancestral origin. Functional analyses demonstrate that both variants disrupt canonical splice sites, resulting in aberrant transcripts presumably subject to nonsense-mediated mRNA decay, consistent with haploinsufficiency as the primary pathogenic mechanism. Clinically, carriers exhibit a relatively late-onset HCM phenotype, with age-dependent penetrance and variable expression. Although recurrent within families and occasionally associated with sudden cardiac death, these variants do not appear to confer a more severe disease course compared with other truncating *MYBPC3* or sarcomeric gene mutations. The identification of asymptomatic individuals within affected pedigrees underscores the clinical utility of genetic testing, not only for confirming diagnosis but also for guiding surveillance and preventive strategies in at-risk relatives.

The identification of these regionally enriched founder variants carries important implications for molecular diagnosis, cascade screening, and clinical management. Moreover, their predictable genetic and geographic distribution highlights their potential as targets for emerging gene-specific therapies, emphasizing the value of integrating population genetics into precision medicine approaches for hereditary cardiomyopathies.

## Figures and Tables

**Table 1:** Comparison of baseline characteristics, disease evolution, and cardiovascular events during follow-up in HCM patients carrying the c.3331-1G>A or c.1458-1G>A variants. * SVT, VF, appropriate ICD activations, and sudden deaths.

Baseline	c.1458-1G>A	c.3331-1G>A	*p*-Value
Number of patients, n	57	34	
Male, n (%)	44 (77%)	22 (65%)	0.2296
Age at diagnosis, median [IQR]	49 [34–56]	48.5 [23–61]	0.9658
Diagnosis for symptoms, n (%)	16 (28%)	10 (29%)	1
Age at first contact at the referring centre, median [IQR]	55 [44–62]	53 [44–62]	0.5896
Family history of SCD, n (%)	4 (7%)	0 (0%)	0.2928
NYHA class III–IV at diagnosis, n (%)	2 (4%)	3 (9%)	0.3582
Unexplained syncope, n (%)	2 (4%)	1 (3%)	1
Obstructive HCM, n (%)	11 (19%)	8 (24%)	0.7904
Myectomy at first contact, n (%)	0 (0%)	0 (0%)	1
ICD at first contact, n (%)	2 (4%)	0 (0%)	0.5267
Major ventricular arrhythmias * at first contact, n (%)	1 (2%)	0 (0%)	1
PM at first contact, n (%)	0 (0%)	0 (0%)	1
MWT, mm, median [IQR]	19 [16–22]	19 [16–21]	0.6887
EDVi, mL/mq, median [IQR]	43 [38–57]	42 [37–47]	0.1235
LVEF < 50%, n (%)	1 (2%)	3 (9%)	0.1450
LAD, mm, median [IQR]	42 [36–48]	42 [40–48]	0.4743
Apical HCM, n (%)	2 (4%)	3 (9%)	0.3582
Apical aneurysm, n (%)	0 (0%)	1 (3%)	0.3736
LVOT obstruction, n (%)	8 (14%)	6 (18%)	0.7657
Medioventricular obstruction, n (%)	3 (5%)	2 (6%)	1
Moderateor severe MR, n (%)	3 (5%)	3 (9%)	0.6675
Restrictive pattern, n (%)	3 (5%)	1 (3%)	1
Follow-up			
Years of follow-up, median [IQR]	9.4 [2.8–14]	8.5 [3–15]	0.8335
Ventricular arrhythmias *, n (%)	3 (5%)	4 (12%)	0.4178
Pacemaker implantation, n (%)	0 (0%)	1 (3%)	0.3736
ICD implantation, n (%)	18 (32%)	11 (32%)	1
Appropriate activations, n (%)	2 (4%)	2 (6%)	0.6278
Myectomy, n (%)	2 (4%)	1 (3%)	1
End-stage evolution, n (%)	6 (11%)	5 (15%)	0.7409
Heart transplant, n (%)	0 (0%)	2 (6%)	0.1370
Death, n (%)	3 (5%)	6 (18%)	0.0740
Cardiovascular death (includingstroke), n (%)	3 (5%)	3 (9%)	0.6675
TIA/nonfatal stroke, n (%)	0 (0%)	2 (6%)	0.1370
Atrial fibrillation, n (%)	14 (25%)	9 (26%)	1

**Table 2:** Patient and echocardiographic characteristics at baseline, disease evolution and cardiovascular events during follow-up in HCM patients carrying the c.1458-1G>A variant compared to a cohort of patients harbouring P/LP variants in other sarcomeric genes (Group C), a cohort of patients with P/LP non-truncating *MYBPC3* variants (Group A), and a cohort of patients carrying other P/LP truncating MYBPC3 variants (Group B) evaluated at our centre between 2001 and 2024.

Baseline	c.1458-1G>A	Other Sarcomeric Variants	*p*-Value *	NT *MYBPC3*	*p-* Value °	T *MYBPC3*	*p-* Value §
Number of patients, n	57	45		38		46	
Male, n (%)	44 (77%)	24 (53%)	0.0193	23 (60%)	0.1083	34 (74%)	0.8179
Age at diagnosis, median [IQR]	49 [34–56]	40 [24–52]	0.0126	43 [25–52]	0.1351	38 [22–42]	0.0000
Diagnosis for symptoms, n (%)	16 (28%)	15 (33%)	0.6657	11 (29%)	1	10 (22%)	0.5017
Age at first contact at the referring centre, median [IQR]	55 [44–62]	48 [34–56]	0.0065	49 [32–61]	0.1254	41 [28–51]	0.0000
Family history of SCD, n (%)	4 (7%)	13 (29%)	0.0061	4 (10%)	0.7094	18 (39%)	0.0001
NYHA class III–IV at diagnosis, n (%)	2 (4%)	5 (11%)	0.2359	1 (3%)	1	3 (6%)	0.6541
Unexplained syncope, n (%)	2 (4%)	5 (11%)	0.2359	5 (13%)	0.1118	0 (0%)	0.5009
Obstructive HCM, n (%)	11 (19%)	15 (33%)	0.1163	11 (29%)	0.3248	11 (24%)	0.6328
Myectomy at first contact, n (%)	0 (0%)	0 (0%)	1	1 (3%)	0.4000	0 (0%)	1
ICD at first contact, n (%)	2 (4%)	4 (9%)	0.4012	3 (8%)	0.3859	4 (9%)	0.4034
Major ventricular arrhythmias * at first contact, n (%)	1 (2%)	1 (2%)	1	4 (10%)	0.1535	1 (2%)	1
PM at first contact, n (%)	0 (0%)	0 (0%)	1	1 (3%)	0.4000	0 (0%)	1
MWT, mm, median [IQR]	19 [16–22]	19 [16–22]	0.8882	18 [15–25]	0.9746	20 [17–27]	0.3533
EDVi, mL/mq, median [IQR]	43 [38–57]	43 [34–57]	0.6106	46 [43–48]	0.8999	39 [32–52]	0.1014
LVEF < 50%, n (%)	1 (2%)	2 (4%)	0.5817	4 (10%)	0.1535	3 (6%)	0.3220
LAD, mm, median [IQR]	42 [36–48]	44 [40–51]	0.0918	45 [36–50]	0.2681	41 [35–48]	0.7482
Apical HCM, n (%)	2 (4%)	2 (4%)	1	4 (10%)	0.2134	2 (4%)	1
Apical aneurism, n (%)	0 (0%)	1 (2%)	0.4412	1 (3%)	0.4000	0 (0%)	1
LVOT obstruction, n (%)	8 (14%)	13 (29%)	0.0854	10 (26%)	0.1822	11 (24%)	0.2135
Medioventricular obstruction, n (%)	3 (5%)	2 (4%)	1	1 (3%)	0.6476	0 (0%)	0.2513
moderate or severe MR, n (%)	3 (5%)	4 (9%)	0.6965	1 (3%)	0.6476	2 (4%)	1
Restrictive pattern, n (%)	3 (5%)	2 (4%)	1	3 (8%)	0.6805	3 (6%)	1
Follow -up							
Years of follow-up, median [IQR]	9.4 [2.8–14]	8.5 [5.2–11.7]	0.6912	9.2 [6.7–14]	0.4931	9.9 [3.4–13.4]	0.8536
Major ventricular arrhythmias *, n (%)	3 (5%)	2 (4%)	1	5 (13%)	0.2596	5 (11%)	0.4616
Pacemaker implantation, n (%)	0 (0%)	0 (0%)	1	1 (3%)	0.4000	2 (4%)	0.1970
ICD implantation, n (%)	18 (32%)	14 (31%)	1	11 (29%)	0.8238	17 (37%)	0.6763
Appropriate activations, n (%)	2 (4%)	2 (4%)	1	2 (5%)	1	3 (6%)	0.6541
Myectomy, n (%)	2 (4%)	9 (20%)	0.010	5 (13%)	0.1118	6 (13%)	0.1349
End-stage evolution, n (%)	6 (11%)	3 (7%)	0.7276	1 (3%)	0.2366	1 (2%)	0.1272
Heart transplant, n (%)	0 (0%)	3 (7%)	0.0826	1 (3%)	0.4000	2 (4%)	0.1970
Death, n (%)	3 (5%)	4 (9%)	0.6965	4 (10%)	0.4321	2 (4%)	1
Cardiovascular death (including stroke), n (%)	3 (5%)	2 (4%)	1	2 (5%)	1	2 (4%)	1
TIA/nonfatal stroke, n (%)	0 (0%)	3 (7%)	0.0878	0 (0%)	1	2 (4%)	0.1970
Atrial fibrillation	14 (25%)	10 (22%)	0.8184	9 (24%)	1	9 (20%)	0.6372

* *p*-value refers to the comparison between c.1458-1G>A and other sarcomeric variants; ° *p*-value refers to the comparison between c.1458-1G>A and non-truncating MYBPC3 variants; § *p*-value refers to the comparison between c.1458-1G>A and other truncating MYBPC3 variants.

**Table 3:** Patient and echocardiographic characteristics at baseline, disease evolution and cardiovascular events during follow-up in HCM patients carrying the c.3331-1G>A variant compared to a cohort of patients harbouring P/LP variants in other sarcomeric genes (Group C), a cohort of patients with P/LP non-truncating *MYBPC3* variants (Group A), and a cohort of patients carrying other P/LP truncating *MYBPC3* variants (Group B) evaluated at our centre between 2001 and 2024.

Baseline	c.3331-1G>A	Other Sarcomeric Variants	*p*-Value *	NT *MYBPC3*	*p* Value °	T *MYBPC3*	*p* Value §
Number of patients, n	34	45		38		46	
Male, n (%)	22 (65%)	24 (53%)	0.3617	23 (60%)	0.8091	34 (74%)	0.4612
Age at diagnosis, median [IQR]	48.5 [23–61]	40 [24–52]	0.0627	43 [25–52]	0.4013	38 [22–42]	0.0017
Diagnosis for symptoms, n (%)	10 (29%)	15 (33%)	0.8089	11 (29%)	1	10 (22%)	0.4474
Age at first contact at the referring centre, median [IQR]	53 [44–62]	48 [34–56]	0.0437	49 [32–61]	0.1254	41 [28–51]	0.0002
Family history of SCD, n (%)	0 (0%)	13 (29%)	0.0004	4 (10%)	0.1168	18 (39%)	0.0001
NYHA class III–IV at diagnosis, n (%)	3 (9%)	5 (11%)	1	1 (3%)	0.3379	3 (6%)	0.6954
Unexplained syncope, n (%)	1 (3%)	5 (11%)	0.2286	5 (13%)	0.2032	0 (0%)	0.4250
Obstructive HCM, n (%)	8 (24%)	15 (33%)	0.4544	11 (29%)	0.7894	11 (24%)	1
Myectomy at first contact, n (%)	0 (0%)	0 (0%)	1	1 (3%)	1	0 (0%)	1
ICD at first contact, n (%)	0 (0%)	4 (9%)	0.1300	3 (8%)	0.2418	4 (9%)	0.1325
Major ventricular arrhythmias * at first contact, n (%)	0 (0%)	1 (2%)	1	4 (10%)	0.1168	1 (2%)	1
PM at first contact, n (%)	0 (0%)	0 (0%)	1	1 (3%)	1	0 (0%)	1
MWT, mm, median [IQR]	19 [16–21]	19 [16–22]	0.8091	18 [15–25]	0.9746	20 [17–27]	0.2631
EDVi, mL/mq, median [IQR]	42 [37–47]	43 [34–57]	0.5473	46 [43–48]	0.8999	39 [32–52]	0.6686
LVEF < 50%, n (%)	3 (9%)	2 (4%)	0.6468	4 (10%)	1	3 (6%)	0.6954
LAD, mm, median [IQR]	42 [40–48]	44 [40–51]	0.4949	45 [36–50]	0.2681	41 [35–48]	0.2090
Apical HCM, n (%)	3 (9%)	2 (4%)	0.6468	4 (10%)	1	2 (4%)	0.6458
Apical aneurysm, n (%)	1 (3%)	1 (2%)	1	1 (3%)	1	0 (0%)	0.4250
LVOT obstruction, n (%)	6 (18%)	13 (29%)	0.2956	10 (26%)	0.4104	11 (24%)	0.5868
Medioventricular obstruction, n (%)	2 (6%)	2 (4%)	1	1 (3%)	0.5992	0 (0%)	0.1775
moderate or severe MR, n (%)	3 (9%)	4 (9%)	1	1 (3%)	0.3379	2 (4%)	0.6458
Restrictive pattern, n (%)	1 (3%)	2 (4%)	1	3 (8%)	0.6167	3 (6%)	0.6329
Follow-up							
Years of follow-up, median [IQR]	8.5 [3–15]	8.5 [5.2–11.7]	0.9063	9.2 [6.7–14]	0.4931	9.9 [3.4–13.4]	0.7052
Major ventricular arrhythmias *, n (%)	4 (12%)	2 (4%)	0.3938	5 (13%)	1	5 (11%)	1
Pacemaker implantation, n (%)	1 (3%)	0 (0%)	0.4304	1 (3%)	1	2 (4%)	1
ICD implantation, n (%)	11 (32%)	14 (31%)	1	11 (29%)	0.8018	17 (37%)	0.8132
Appropriate activations, n (%)	2 (6%)	2 (4%)	1	2 (5%)	1	3 (6%)	1
Myectomy, n (%)	1 (3%)	9 (20%)	0.0373	5 (13%)	0.2032	6 (13%)	0.2289
End-stage evolution, n (%)	5 (15%)	3 (7%)	0.2798	1 (3%)	0.0940	1 (2%)	0.0782
Heart transplant, n (%)	2 (6%)	3 (7%)	1	1 (3%)	0.5992	2 (4%)	1
Death, n (%)	6 (18%)	4 (9%)	0.3133	4 (10%)	0.5008	2 (4%)	0.0662
Cardiovascular death (including stroke), n (%)	3 (9%)	2 (4%)	0.6468	2 (5%)	0.6618	2 (4%)	0.6458
TIA/nonfatal stroke, n (%)	2 (6%)	3 (7%)	1	0 (0%)	0.2332	2 (4%)	1
Atrial fibrillation	9 (26%)	10 (22%)	0.7915	9 (24%)	0.7928	9 (20%)	0.5896

* *p*-value refers to the comparison between c.1458-1G>A and other sarcomeric variants; ° *p*-value refers to the comparison between c.1458-1G>A and non-truncating *MYBPC3* variants; § *p*-value refers to the comparison between c.1458-1G>A and other truncating *MYBPC3* variants.

## Data Availability

Raw data are available at the following link: https://doi.org/10.5281/zenodo.17717237.

## Supplementary Materials

The following supporting information can be downloaded at: https://www.mdpi.com/article/10.3390/genes17080882/s1, Supplementary Table S1. STSs tested and genotypes identified in patients carrying the c.1458-1G>A variant; Table S2. STSs tested and genotypes identified in patients carrying the c.3331-1G>A variant.

## Abbreviations

The following abbreviations are used in this manuscript:

HCM	Hypertrophic cardiomyopathy
ACMG	American College of Medical Genetics and Genomics
LV	Left ventricular
RVH	Right ventricular hypertrophy
LVH	Left ventricular hypertrophy
HF	Heart failure
SCD	Sudden cardiac death
NMD	Nonsense-mediated mRNA decay
MLVWT	Maximum left ventricular wall thickness
ECG	Electrocardiogram
CMR	Cardiac magnetic resonance
NGS	Next-generation sequencing
MAF	Minor allele frequency
RNA-Seq	RNA sequencing
TPM	Transcripts per million
UCSC	University of California, Santa Cruz
FAM	6-fluorescein amidite
SNP	Single nucleotide polymorphism
LVOT	Left ventricular outflow tract
LVEF	Left ventricular ejection fraction
ICD	Implantable cardioverter–defibrillator
