# Supplementary material for: Clinical, Transcriptional and Haplotype Characterization of Recurrent MYBPC3 Splice-Site Variants c.1458-1G>A and c.3331-1G>A Associated with Hypertrophic Cardiomyopathy in Northern Italy
Source: Genes (Basel). 2026 Jul 28;17(8):882. doi: 10.3390/genes17080882 (PMC13512870; doi:10.3390/genes17080882)
Supplement: Supplementary file 1 [file genes-17-00882-s001.zip › genes-4374046-supplementary.pdf]

**GRCh38 hg38**	**D11S1993 Chr11:43588370-43588779**	**D11S986 Chr11:44700564-44700838**	**D11S4174 Chr11:45236466-45236824**	**D11S1344 Chr11:46145309-46145675**	***MYBPC3*** **Chr11:Chr11:47342745**	**D11S1395 Chr11:54736244-54736497**
13BO4	239-239	175-171	294-294	298-300	c.1458-1G>A	240-240
27BO1	239-245	175-173	294-294	300-300	c.1458-1G>A	240-240
20FE1	239-260	175-155	294-292	300-300	c.1458-1G>A	240-244
11FE1	239-257	175-179	294-294	290-300	c.1458-1G>A	240-244
13FE1	239-239	175-163	294-292	300-300	c.1458-1G>A	240-244
4FE1	239-245	175-171	294-298	292-300	c.1458-1G>A	240-244
12FE1	239-239	175-153	294-298	290-300	c.1458-1G>A	240-240
15FE1	239-254	175-181	294-288	290-300	c.1458-1G>A	240-244
17FE2	239-245	175-173	294-294	300-300	c.1458-1G>A	240-244
22FE1	239-257	175-188	294-294	290-300	c.1458-1G>A	240-244
26FE1	239-239	175-177	294-294	300-300	c.1458-1G>A	240-244
11BO4	239-239	153-153	294-298	300-300	c.1458-1G>A	240-240
1BO1	239-239	153-153	294-294	300-300	c.1458-1G>A	240-244
2BO1	245-245	155-175	294-292	292-300	c.1458-1G>A	240-240
3BO2	239-254	153-155	294-299	302-300	c.1458-1G>A	240-244
4BO1	254-254	175-171	294-294	306-300	c.1458-1G>A	240-244
11BO1	239-239	153-171	294-299	300-300	c.1458-1G>A	240-244
18FE1	254-260	153-169	294-294	300-300	c.1458-1G>A	240-244
6BO1	239-257	153-153	294-298	302-300	c.1458-1G>A	240-244
7BO1	239-254	153-153	294-294	298-300	c.1458-1G>A	240-240
8BO1	239-245	153-157	294-292	290-300	c.1458-1G>A	240-244
9BO1	254-254	153-173	294-292	290-300	c.1458-1G>A	240-244
10BO1	254-254	153-173	294-292	298-300	c.1458-1G>A	240-244
6FE2	254-254	153-173	294-298	298-300	c.1458-1G>A	240-244
21FE1	239-254	153-173	294-298	298-300	c.1458-1G>A	240-240
19FE1	254-257	151-175	294-292	290-300	c.1458-1G>A	240-244
14FE1	254-257	175-179	294-294	298-300	c.1458-1G>A	240-244
5FE1	250-254	171-175	294-294	302-300	c.1458-1G>A	240-240
9FE1	243-245	153-181	294-294	302-300	c.1458-1G>A	240-244
16FE1	245-254	171-175	294-288	290-300	c.1458-1G>A	240-240

Supplementary Table S1. STSs tested and genotypes identified in patients carrying the c.1458-1G>A variant.

**GRCh38 hg38**	**D11S1993 Chr11:43588370-43588779**	**D11S986 Chr11:44700564-44700838**	**D11S4174 Chr11:45236466-45236824**	**D11S1344 Chr11:46145309-46145675**	***MYBPC3* Chr11:Chr11:47332974**	**D11S1395 Chr11:54736244-54736497**
89BO	254-256	153-153	298-311	294-298	c.3331-1G>A	240-244
2FE1	254-245	153-177	298-311	294-294	c.3331-1G>A	240-240
15BO	254-245	153-163	295-311	294-294	c.3331-1G>A	240-244
19BO	254-245	153-165	295-311	294-298	c.3331-1G>A	240-244
33BO	254-256	153-171	293-311	294-290	c.3331-1G>A	240-240
20BO	254-254	153-175	298-311	294-298	c.3331-1G>A	240-244
25FE1	254-254	153-155	298-311	294-298	c.3331-1G>A	240-240
17BO1	239-242	153-153	283-311	294-300	c.3331-1G>A	240-244
18BO1	239-245	153-167	293-311	294-296	c.3331-1G>A	240-248
16BO1	239-242	171-173	288-311	294-298	c.3331-1G>A	240-244
8FE1	239-245	173-177	293-311	294-300	c.3331-1G>A	240-244
7FE1	239-245	153-153	293-311	294-300	c.3331-1G>A	240-244

Supplementary Table S2. STSs tested and genotypes identified in patients carrying the c.3331-1G>A variant.
